# Exploring the role of splicing in *TP53* variant pathogenicity through predictions and minigene assays

**DOI:** 10.1186/s40246-024-00714-5

**Published:** 2025-01-08

**Authors:** Cristina Fortuno, Inés Llinares-Burguet, Daffodil M. Canson, Miguel de la Hoya, Elena Bueno-Martínez, Lara Sanoguera-Miralles, Sonsoles Caldes, Paul A. James, Eladio A. Velasco-Sampedro, Amanda B. Spurdle

**Affiliations:** 1 Population Health Program, QIMR Berghofer Medical Research Institute, Herston, QLD 4006 Australia; 2 Splicing and Genetic Susceptibility to Cancer, Instituto de Biomedicina y Genética Molecular de Valladolid (IBGM), Consejo Superior de Investigaciones Científicas - Universidad de Valladolid (CSIC-UVa), 47003 Valladolid, Spain; 3 Molecular Oncology Laboratory, Hospital Clínico San Carlos, IdISSC (Instituto de Investigación Sanitaria del Hospital Clínico San Carlos), Madrid, Spain; 4 Parkville Familial Cancer Centre, Peter MacCallum Cancer Centre and Royal Melbourne Hospital, Melbourne, VIC Australia; 5 Sir Peter MacCallum Department of Oncology, University of Melbourne, Melbourne, VIC Australia; 6 Faculty of Medicine, The University of Queensland, Brisbane, QLD 4006 Australia

**Keywords:** TP53, Splicing, SpliceAI, PVS1, VCEP specifications

## Abstract

**Background:**

*TP53* variant classification benefits from the availability of large-scale functional data for missense variants generated using cDNA-based assays. However, absence of comprehensive splicing assay data for *TP53* confounds the classification of the subset of predicted missense and synonymous variants that are also predicted to alter splicing. Our study aimed to generate and apply splicing assay data for a prioritised group of 59 *TP53* predicted missense or synonymous variants that are also predicted to affect splicing by either SpliceAI or MaxEntScan.

**Methods:**

We conducted splicing analyses using a minigene construct containing *TP53* exons 2 to 9 transfected into human breast cancer SKBR3 cells, and compared results against different splice prediction methods, including correlation with the SpliceAI-10k calculator. We additionally applied the splicing results for *TP53* variant classification using an approach consistent with the ClinGen Sequence Variant Interpretation Splicing Subgroup recommendations.

**Results:**

Aberrant transcript profile consistent with loss of function, and for which a PVS1 (RNA) code would be assigned, was observed for 42 (71%) of prioritised variants, of which aberrant transcript expression was over 50% for 26 variants, and over 80% for 15 variants. Data supported the use of SpliceAI ≥ 0.2 cutoff for predicted splicing impact of *TP53* variants. Prediction of aberration types using SpliceAI-10k calculator generally aligned with the corresponding assay results, though maximum SpliceAI score did not accurately predict level of aberrant expression. Application of the observed splicing results was used to reclassify 27/59 (46%) test variants as (likely) pathogenic or (likely) benign.

**Conclusions:**

In conclusion, this study enhances the integration of splicing predictions and provides splicing assay data for exonic variants to support *TP53* germline classification.

**Graphical abstract:**

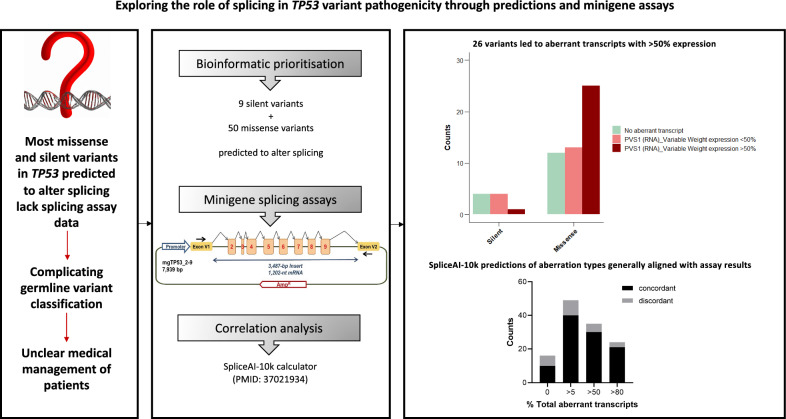

**Supplementary Information:**

The online version contains supplementary material available at 10.1186/s40246-024-00714-5.

## Background

Pathogenic germline variants in the *TP53* gene, known for its critical role in tumor suppression, cause the multi-cancer Li-Fraumeni syndrome that is associated with a very high risk of cancer [1]. Most known germline pathogenic variants identified in *TP53* encode missense substitutions, causing a dominant-negative effect, loss-of-function, or gain-of-function, thereby compromising the tumor-suppressive activity of the p53 protein. While pathogenic variants targeting splice site dinucleotide positions have been described, variant-induced aberrant splicing does not appear to represent the most common mechanism of pathogenicity for *TP53*.

Recently, the ClinGen Sequence Variant Interpretation (SVI) Splicing Subgroup developed recommendations to promote standardised approaches to interpreting splicing bioinformatic and assay data for variant interpretation [2]. Calibration analyses indicated that SpliceAI [3] had sufficient accuracy for use in predicting variant impact on splicing in variant curation, and many ClinGen Variant Curation Expert Panels (VCEPs) have now incorporated use of this tool in their criteria specifications. This includes the *TP53* v2 specifications, where SpliceAI replaced MaxEntScan (MES) [4, 5] as the nominated splice prediction tool. When a variant predicted to encode a missense substitution is also predicted to alter splicing, it complicates application of bioinformatic tools and functional assays that capture only protein level effects. For *TP53* specifically, this would mean that computational codes should not be assigned based on the Align-GVGD and BayesDel missense bioinformatic prediction tools [6], and that the use of Kato [7], Giacomelli [8], and other cDNA-based functional assay results cannot be employed for the purpose of applying functional evidence codes. Similarly, synonymous substitution variants predicted to alter splicing need additional splicing and/or clinical data to shift classification outside of the VUS category.

A major challenge in investigating *TP53* variants predicted to impact splicing is the scarcity of publicly available splicing assays to confirm predictions, especially for variants outside the splice site dinucleotide donor and acceptor positions. This scarcity is underscored by our literature review of *TP53* splicing assays from PubMed studies and ClinVar submissions (conducted in 2020, Supplementary Table 1). Of 89 unique *TP53* variants with published RNA data (only eight variants annotated as synonymous, and five as predicted missense), splicing assay results for 34 (38%) remain unrepresented in two public splicing-related databases: the MutSpliceDB RNA-seq data resource with 73 *TP53* entries (mostly splice site dinucleotide variants) [9]; and the SpliceVarDB database of published splicing assay results generated using diverse methodologies, which currently contains information on 79 *TP53* variants (~ 25% exonic) [10].

Our study aimed to address this challenge by investigating a prioritised group of predicted splice-impacting *TP53* variants, including apparent synonymous substitutions outside splice regions and missense variants, which have traditionally not been examined for their effects on splicing. Through this investigation, we sought to overall refine the use of splicing data within *TP53* germline classification guidelines, and to assist with the accurate classification of specific variants using new data generated.

## Methods

### Variant prioritisation

We performed SpliceAI predictions for every *TP53* exonic single nucleotide substitution of the protein coding sequence in relation to the MANE Select transcript (NM_000546.6). From the total 3,547 variants, we selected 38 apparent synonymous outside splice regions (as defined by Ensembl Variant Effect Predictor i.e. first and last three nucleotides of the exon) and missense variants in exons 2 to 9 with a SpliceAI raw maximum delta score ≥ 0.5 using a 10 kb analysis window, the cutoff originally suggested by developers for the prediction of cryptic splice sites [3]. We also selected an additional group of 21 predicted synonymous outside splice regions and missense variants in exons 2 to 9 with a “High” prediction to alter splicing through either native loss (n = 14) or donor gain (n = 7) by MES (no data available for acceptor gain), as previously defined [11], but with SpliceAI < 0.5; 10 of these variants had SpliceAI score ≥ 0.2, the cutoff currently used within the *TP53* specifications v2 as recommended by the SVI Splicing group [2]. This resulted in a total of 59 prioritised test variants: nine synonymous variants (all outside exonic splice regions) and 50 missense variants (of which 24 were inside the splice regions).

We additionally selected one negative control (wild-type sequence), and eight positive control variants located at donor or acceptor splice site dinucleotide positions with existing splicing data available in databases and/or the literature. Positive control variants were located in introns 3, 4, 5, 6, 7 or 8. All 8 positive control variants were classified in ClinVar as pathogenic or likely pathogenic, and predicted to alter splicing by both SpliceAI and MES.

### Minigene construct design and site-directed mutagenesis, and splicing assays

Methods followed the same approach as detailed elsewhere (Canson et al., under review) [12]. In brief, a 3487-bp fragment (Supplementary Fig. 1), including exons 2 to 9 of the *TP53* gene, was synthesised and cloned into the pSAD splicing vector to generate the minigene construct (mgTP53_2-9) [13]. The expected size of the minigene full-length (FL) transcript was 1202-nt. Candidate variants were introduced into the wild-type construct by site-directed mutagenesis using the QuikChange Lightning Kit (Agilent, Santa Clara, CA, USA) (mutagenesis primers in Supplementary Table 2) and were confirmed by sequencing (Macrogen, Madrid, Spain). About 2 × 10^5^ of human breast cancer SK-BR-3 cells (ATCC HTB-30; LGC Standards, Barcelona, Spain) were grown in 0.5 mL of medium (Minimum Essential Medium -MEM-, 10% Fetal Bovine Serum, 1% nonessential amino acids, 2 mM Glutamine and 1% Penicillin/Streptomycin; Sigma-Aldrich, St. Louis, MO, USA) in 4-well plates (Nunc, Roskilde, Denmark). Cells were transfected with 1 µg of the wild-type and mutant minigenes using 2µL of lipofectamine LTX (Life Technologies, Carlsbad, CA, USA), followed by a 4-h incubation with cycloheximide (Sigma-Aldrich, St. Louis, MO, USA) to inhibit nonsense-mediated decay. RNA was isolated using the Genematrix Universal RNA Purification Kit (EURx, Gdansk, Poland), with on-column DNAse I digestion. Reverse transcription polymerase chain reaction (RT-PCR) was performed on 400 ng of RNA with the RevertAid First Strand cDNA Synthesis Kit (Life Technologies) using the vector-specific primer RT-PSPL3-RV (5′-TGAGGAGTGAATTGGTCGAA-3′). Wild-type and variant cDNAs were amplified with Platinum Taq DNA polymerase (Life Technologies) and primers SD6-PSPL3_RTFW (5'-TCACCTGGACAACCTCAAAG-3') and RTpSAD-RV (Patent P201231427, CSIC), under the following thermocycling conditions: 94 °C 2 min + 35 cycles x [94 °C, 30 s/59 °C, 30 s/72 °C, 1 min/kb] + 72 °C, 5 min. RT-PCR products were sequenced by Macrogen. Semi-quantitative fluorescent RT-PCRs (26 cycles) were conducted in triplicate to estimate transcript proportions by fragment analysis (Macrogen). Mean peak areas of each transcript (for heights with ≥ 100 relative fluorescence units) and standard deviations were calculated.

### PVS1 (RNA)_variable weight codes assignation to transcripts

Aberrant transcripts (i.e., not FL) produced by each variant were categorised into premature termination codon (PTC) and in-frame, with some variants also producing uncharacterised transcripts at a very low expression (< 3.3%) that was not considered relevant for the purpose of impacting *TP53* function. Based on our splicing assay results, we assigned PVS1 (RNA)_Variable weight codes to all aberrant transcripts observed, following the *TP53* PVS1 flowchart from the VCEP specifications v2. For certain transcripts whose interpretation was not straightforward using the flowchart, specifically in-frame deletions of a few amino acids, we followed the same rationale used by the *TP53* VCEP in developing the PVS1 flowchart. In particular, we applied PVS1 if the in-frame region deleted included any missense variant classified as pathogenic by the *TP53* VCEP. If there was only convincing functional data [7, 8, 14] for any of the amino acids involved suggesting a damaging effect, we applied PVS1_Strong. If there were only bioinformatic predictions for a damaging effect of amino acids in the deleted region, we applied PVS1_Supporting. If the deletions were predicted to be benign, and in the absence of other evidence, we applied PVS1_NA.

### Analysis of splicing predictor pathogenicity performance

We compared the effectiveness of each predictor in identifying PVS1-aberrant transcripts at varying expression levels by analysing the proportions across different transcript groups. In order to compare the proportion of true positive and true negative results, we considered variants producing only FL transcripts at 100% as “no splice impact”, and those yielding PVS1-assigned transcripts at any weight with over 80% expression as “high splice impact”. This was selected as an operational threshold, based on recommendations for level of splice impact used by the ClinGen *BRCA1* and *BRCA2* VCEP (https://cspec.genome.network/cspec/ui/svi/doc/GN092) [15].

Performance for SpliceAI was assessed using binary cutoffs (< 0.5 and ≥ 0.5), and current VCEP-specified used cutoffs (≤ 0.1 and ≥ 0.2) for SpliceAI, and “Low” or no prediction against “High” prediction for MES.

### Correlation between maximum SpliceAI score and level of impact on splicing

We aimed to identify any correlation between SpliceAI scores and expression of aberrant transcripts observed for all test variants assayed, by conducting a Pearson’s correlation test, using SpliceAI maximum delta scores and the total expression of aberrant transcripts that had been assigned a PVS1 (RNA)_Variable weight code. Additionally, we compared the distribution of PVS1-assigned aberrant transcript expression among *TP53* variants with the following SpliceAI score groups: 0–0.2, 0.2–0.5, 0.5–0.8, 0.8–1. We used the Kruskal–Wallis test to determine significant differences in PVS1-assigned aberrant transcript expression across the four SpliceAI score groups, followed by Wilcoxon tests for pairwise comparisons.

### Correlation between SpliceAI-10k calculator prediction of aberration types and assay-detected splice alterations

We utilised the SpliceAI-10k calculator (SAI-10k-calc) [16], an algorithm that interprets the SpliceAI scores to predict specific types of splicing aberrations for each variant assayed. Aberrant transcript predictions were obtained using default settings. We then compared SAI-10k-calc predictions with the observed assay results.

### *TP53* variant reclassifications

We retrieved the ClinVar classification for each *TP53* test variant (as at October 2024). We independently classified each variant according to the *TP53* VCEP specifications v2, following either the missense/synonymous or splicing pathways depending on the *TP53* splicing assay results, into the Pathogenic (P), Likely pathogenic (LP), VUS, Likely benign (LB), and Benign (B) classes.

For the purpose of variant classifications performed in this study, we conservatively applied PVS1 (RNA)_Variable weight only to variants whose aberrant PVS1-assigned transcripts accounted for more than 80% of the total expression, a threshold set for VCEP classification of *BRCA1* and *BRCA2* variants [15]. For synonymous variants in which FL expression was 100%, we applied BP7 (RNA)_Strong in agreement with *TP53* specifications v2.

To assist with the classifications, we searched the *TP53* Database [17], FLOSSIES (https://whi.color.com/), and gnomAD v4.1 [18] for relevant clinical and population data. Other evidence used included the cancerhotspots.org database [19] for the application of PM1_Variable weight, and supplementary material of the *TP53*-clonal hematopoiesis study of *TP53* [20] for the application of PP4_Variable weight.

## Results

### Splicing assay results

A summary of the number of positive predictions and splicing assay results for the selected *TP53* variants encoding synonymous and missense substitutions, as well as the positive and negative control variants, is shown in Table 1. Variant-specific results with all transcripts produced are detailed for variants based on predicted effect and location, as follows: splice site positive controls (Table 2), synonymous variants outside splice regions (Table 3), missense variants outside splice regions (Table 4), missense variants inside splice regions (Table 5). Electropherograms can be visualised in Supplementary Fig. 2.

**Table 1:** Summary of splicing assay results according to test variant groups and controls

Variant group (n total)	SpliceAI		MES	Variants with full-length expression 100%	Variants (n) with aberrant transcript(s) detected	Aberrant transcript(s) meets PVS1 (RNA)_Variable weight (any expression level)	PVS1 (RNA)_Variable weight transcript(s) total expression > 50%	PVS1 (RNA)_Variable weight transcript(s) total expression > 80%
	0.2–0.5	≥ 0.5						
Negative control (1)	NA	NA	NA	1	0	0	0	0
Positive controls (8)	0	8	8	0	8	8	8	8
Synonymous outside splice regions (9)	1	7	3	4	5	5	1	0
Missense outside splice regions (24)	1	19	8	2 (+ 4 uncharacterised transcripts only)	18	18	12	7
Missense inside splice regions (26)	8	12	24	6	20	19	13	8

MES = MaxEntScan

Variants in all variant groups produced aberrant transcripts (i.e. not FL) for which PVS1 (RNA)_Variable weight was assigned (summarised in Table 1). The proportion of variants producing aberrant transcripts was highest for positive control variants located at splice site dinucleotide positions and expected to have high impact (8/8), and lowest for synonymous variants.

#### Negative and positive controls

The wild-type minigene construct mgTP53_2-9 produced the expected FL transcript of 1,202 nucleotides with a 100% expression. All eight splice site dinucleotide positive control variants produced aberrant transcripts, providing 0% of FL transcript and variable percentages of PTC and/or in-frame transcripts, with three variants additionally producing an extremely low level of uncharacterised transcripts (Tables 1 and 2).

**Table 2:** Splicing assay results with corresponding assigned PVS1 (RNA)_Variable weight for the splice site positive controls

Transcript variant (NM_000546.6)	MES (native loss)	SAI	Full-length transcript expression	PTC transcript [expression]	In-frame transcript [expression]	Uncharacterised transcript [expression]	PVS1 (RNA)_Variable Weight (total expression)
c.783-1G > A	HIGH	1	–	▼(I7) [1.6% ± 0.2%]	△(E8p24) [48.2% ± 1.4%] ▼(E8p3) [50.2% ± 1.6%]	–	PVS1_Strong (48.2%), PVS1 (1.6%), and PVS1_NA (50.2%)
c.782 + 1G > A	HIGH	0.99	–	△(E7) [10.3% ± 1%] ▼(I7) [89.7% ± 1%]	–	–	PVS1 (100%)
c.673-1G > A	HIGH	1	–	▼(E7p49) [63.4% ± 0.7%] △(E7p1) [21.5% ± 0.4%] ▼(I6) [7.7% ± 1.1%]	–	320-nt [2.5% ± 0.2%] 1050-nt [3.5% ± 0.1%] 1155-nt [1.4% ± 0.3%]	PVS1 (92.6%)
c.672 + 1G > A	HIGH	0.99	–	▼(E6q5) [89.9% ± 0.3%] △(E6) [5.8% ± 0.1%]	–	925-nt [2.3% ± 0.1%] 1005-nt [2.1% ± 0.2%]	PVS1 (95.7%)
c.560-2A > C	HIGH	0.98	–	△(E6p17) [78.2% ± 1.3%] △(E6) [16.6% ± 0.9%] ▼(I5) [2.6% ± 0.2%]	–	–	PVS1 (100%)
c.559 + 1G > A	HIGH	1	–	Δ(E5q46) [90.9% ± 0.2%] ▼(I5) [5.9% ± 0.2%]	-	953-nt [3.3%]	PVS1 (96.8%)
c.376-1G > A	HIGH	1	–	–	Δ(E5p21) [100%]	–	PVS1 (100%)
c.97-1G > A	HIGH	0.99	–	△(E4p19) [15.3% ± 0.8%]	△(E4) [84.7% ± 0.8%]	–	PVS1 (100%)

MES = MaxEntScan; PTC = Premature termination codon; SAI = SpliceAI

All positive controls met PVS1 (RNA)_Variable weight based on *TP53* specifications v2 with an expression higher than 92.6%, except for c.783-1G > A. For this variant, half of the abnormal expression (50.2%) corresponded to a very short in-frame transcript ▼(E8p3) (p.Ser261_Gly262insSer) with no obvious damaging impact and benign impact prediction using MutationTaster (no prediction available using BayesDel), for which we subsequently assigned PVS1_NA. The other two transcripts produced by this control variant, △(E8p24) [48.2%] and ▼(I7) [1.6%], were assigned PVS1_Strong and PVS1, respectively.

#### Apparent synonymous variants outside splice regions

Of the nine synonymous variants assessed, four had FL transcript expression at 100%, and an aberrant transcript meeting PVS1 at any weight was observed for the remaining five variants (Tables 1 and 3).

**Table 3:** Splicing assay results with corresponding assigned PVS1 (RNA)_Variable weight for the synonymous variants outside splice regions

Transcript variant (NM_000546.6)	Protein variant (NP_000537.3)	MES (donor gain)	SAI	Full-length transcript expression	PTC transcript [expression]	In-frame transcript [expression]	Uncharacterised transcript	PVS1 (RNA)_Variable Weight (total expression)
c.879G > T	p.Gly293 =	HIGH	0.99	77.2% ± 4.2%	–	Δ(E8q42) [22.8% ± 4.2%]	–	PVS1_Strong (22.8%)
c.837G > C	p.Gly279 =	LOW	0.71	100%	–	–	–	NA
c.837G > T	p.Gly279 =	LOW	0.5	100%	–	–	–	NA
c.834 T > A	p.Pro278 =	LOW	0.92	94.7% ± 0.1%	Δ(E8p53) [5.3% ± 0.1%]	–	–	PVS1 (5.3%)
c.816G > A	p.Val272 =	HIGH	0.02	100%	–	–	–	NA
c.597A > T	p.Gly199 =	HIGH	0.42	33% ± 0.5%	△(E6q77) [55% ± 0.5%] △(E6) [10.3% ± 0.4%] ▼(E6q5) [1.6% ± 0.8%]	–	–	PVS1 (66.9%)
c.516 T > A	p.Val172 =	LOW	0.5	73.8% ± 3.3%	Δ(E5q46) [26.2% ± 3.3%]		–	PVS1 (26.2%)
c.315C > T	p.Gly105 =	LOW	0.73	84.1% ± 1.0%	△(E4q200) [13.0% ± 0.4%] Δ(E4q62) [3.0% ± 0.6%]		–	PVS1 (16%)
c.114A > G	p.Gln38 =	LOW	0.61	100%	–	–	–	NA

MES = MaxEntScan; PTC = Premature termination codon; SAI = SpliceAI

Only the c.597A > T variant (absent in ClinVar) led to an aberrant transcript expression level higher than 50% (66.9%), based on three transcripts which all met PVS1. The remaining four variants each led to the expression aberrant transcripts, with total level ranging from 5.3% to 22.8%, and PVS1 (RNA) annotation either PVS1_Strong (c.879G > T only) or PVS1.

#### Apparent missense variants outside splice regions

Of the 24 missense variants outside splice regions, 18 led to an aberrant transcript meeting PVS1 at any weight, four missense produced only uncharacterised transcripts at extremely low level (< 3.3%), and the remaining two had FL transcript expression at 100% (Tables 1 and 4).

**Table 4:** Splicing assay results with corresponding assigned PVS1 (RNA)_Variable weight for the missense variants outside splice regions

Transcript variant (NM_000546.6)	Protein variant (NP_000537.3)	MES (donor gain)	SAI	Full-length transcript expression	PTC transcript [expression]	In-frame transcript [expression]	Uncharacterised transcript [expression]	PVS1 (RNA)_Variable Weight (total expression)
c.923T > A	p.Leu308Gln	LOW	0.96	84.5% ± 1.4%	Δ(E9p5) [15.5% ± 1.4%]	–	–	PVS1 (15.5%)
c.835G > A	p.Gly279Arg	LOW	0.92	70% ± 0.3%	–	Δ(E8p54) [30% ± 0.3%]	–	PVS1 (30%)
c.817C > A	p.Arg273Ser	HIGH	0.05	100%	–	–	–	NA
c.812A > C	p.Glu271Ala	HIGH	0.01	97% ± 0.3%	–	–	955-nt [3% ± 0.3%]	NA
c.811G > A	p.Glu271Lys	HIGH	0.01	97.3% ± 0.4%	–	–	955-nt [2.7% ± 0.4%]	NA
c.811G > C	p.Glu271Gln	HIGH	0.32	96.7% ± 0.1%	–	–	955-nt [3.3% ± 0.1]	NA
c.797G > T	p.Gly266Val	HIGH	0.17	98.4% ± 0.3%	–	–	955-nt [1.6 ± 0.3%]	NA
c.710T > G	p.Met237Arg	LOW	0.58	95.2% ± 0.3%	△(E7p38) [4.8% ± 0.3%]	–	–	PVS1 (4.8%)
c.551A > G	p.Asp184Gly	LOW	0.98	37.8% ± 0.6%	–	Δ(E5q9) [62.2% ± 0.6%]	–	PVS1_Supporting (62.2%)
c.410T > A	p.Leu137Gln	LOW	0.7	-	–	△(E5p36) [100%]	–	PVS1 (100%)
c.368C > G	p.Thr123Ser	LOW	0.95	59.9% ± 0.7%	–	Δ(E4q12) [40.1% ± 0.7%]	–	PVS1_Strong (40.1%)
c.362C > A	p.Ser121Tyr	LOW	0.89	82.8% ± 0.2%	Δ(E4q16) [17.2% ± 0.2%]	–	–	PVS1 (17.2%)
c.359A > G	p.Lys120Arg	LOW	0.93	17.5% ± 0.8%	Δ(E4q16) [67.5% ± 0.3%] Δ(E4q16)Δ(E5) [6.4% ± 0.2%]	–	364-nt [8.6% ± 0.8%]	PVS1 (73.9%)
c.356C > G	p.Ala119Gly	LOW	0.82	5.1% ± 0.3%	Δ(E4q20) [24.0% ± 2.7%] Δ(E4q200) [70.9% ± 2.7%]	–	–	PVS1 (94.9%)
c.325T > A	p.Phe109Ile	LOW	0.64	100%		–	–	NA
c.318C > G	p.Ser106Arg	HIGH	1	–	Δ(E4q58) [100%]		–	PVS1 (100%)
c.314G > T	p.Gly105Val	LOW	0.98	13.6% ± 0.5%	–	△(E4q63) [86.4% ± 0.5%]	–	PVS1_Strong (86.4%)
c.236C > G	p.Ala79Gly	LOW	0.8	45.8% ± 0.4%	△(E4q140) [54.2% ± 0.4%]		–	PVS1 (54.2%)
c.206C > T	p.Ala69Val	LOW	0.61	34.5% ± 0.5%	△(E4q200) [3.5%]	△(E4q171) [62.1% ± 0.5%]	–	PVS1_Strong (62.1%) and PVS1 (3.5%)
c.182A > G	p.Asp61Gly	LOW	0.98	11.8% ± 0.3%	Δ(E4q194) [88.2% ± 0.3%]	–	–	PVS1 (88.2%)
c.178C > A	p.Pro60Thr	HIGH	0.67	7.4% ± 1.4%	△(E4q200) [90.5% ± 1.7%]	–	887-nt [2.1% ± 0.3%]	PVS1 (90.5%)
c.178C > G	p.Pro60Ala	LOW	0.5	67.9% ± 1.5%	△(E4q200) [32.1% ± 1.5%]	–	–	PVS1 (32.1%)
c.50A > T	p.Glu17Val	HIGH	0.99	–	△(E2q26) [92.3% ± 1.3%]	–	1063-nt [2.2% ± 0.3%] 973-nt [5.5% ± 1.0%]	PVS1 (92.3%)
c.46C > A	p.Gln16Lys	LOW	0.84	47% ± 0.7%	△(E2q31) [53% ± 0.7%]	–	–	PVS1 (53%)

MES = MaxEntScan; PTC = Premature termination codon; SAI = SpliceAI

Of the 18 variants for which PVS1 was assigned, 12 variants produced aberrant transcripts with a total expression higher than 50%, in which ten had PVS1 assigned at full strength. Of these 12, seven had an expression higher than 80%, of which two had a full expression of 100%.

#### Apparent missense variants inside splice regions

Of the 26 missense variants inside splice regions, an aberrant transcript was observed in 20 variants, with the remaining six having FL transcript expression at 100% (Tables 1 and 5).

**Table 5:** Splicing assay results with corresponding assigned PVS1 (RNA)_Variable weight for the missense variants inside splice regions

Transcript variant (NM_000546.6)	Protein variant (NP_000537.3)	MES (native loss)	SAI	Full-length transcript expression	PTC transcript [expression]	In-frame transcript [expression]	Uncharacterised transcript [expression]	PVS1 (RNA)_Variable Weight (total expression)
c.993G > C	p.Gln331His	HIGH	0.64	20.2% ± 0.6%	▼(I9-mg) [15% ± 0.8%] △(E9) [61.5% ± 1.2%]	–	773-nt [3.3% ± 0.2%]	PVS1 (76.5%)
c.993G > T	p.Gln331His	HIGH	0.62	25.3% ± 4,9%	▼(I9-mg) [29.7% ± 6.5] △(E9) [45% ± 1.5%]	–	–	PVS1 (74.7%)
c.992A > C	p.Gln331Pro	HIGH	0.33	62.4% ± 8.5%	▼(I9-mg) [37.6% ± 8.5%]	–	–	PVS1 (37.6%)
c.992A > G	p.Gln331Arg	HIGH	0.36	70.6% ± 0.5%	▼(I9-mg) [19.5% ± 0.6%] △(E9) [9.9% ± 0.1%]	–	–	PVS1 (29.4%)
c.992A > T	p.Gln331Leu	HIGH	0.35	70.8% ± 1.7%	▼(I9-mg) [24.5% ± 1.9] △(E9) [4.7% ± 0.1%]	–	–	PVS1 (29.2%)
c.922C > G	p.Leu308Val	LOW	0.99	-	–	Δ(E9p3) [100%]	–	PVS1_NA (100%)
c.919G > T	p.Ala307Ser	HIGH	0.16	100%	–	–	–	NA
c.784G > A	p.Gly262Ser	MODERATE*	0.52	100%	–	–	–	NA
c.782G > A	p.Ser261Asn	HIGH	0.27	57.6% ± 1.8%	△(E7) [2.6% ± 0.3%] ▼(I7) [39.8% ± 2.1%]	–	–	PVS1 (42.4%)
c.782G > C	p.Ser261Thr	HIGH	0.64	16.6% ± 1.3%	△(E7) [5.7% ± 0.4%] ▼(I7) [77.7% ± 1.6%]	–	–	PVS1 (83.4%)
c.782G > T	p.Ser261Ile	HIGH	0.53	11.8% ± 1.4%	△(E7) [30.3% ± 0.5%] ▼(I7) [57.9% ± 0.9%]	–	–	PVS1 (88.2%)
c.781A > C	p.Ser261Arg	HIGH	0.03	100%	–	–	–	NA
c.781A > G	p.Ser261Gly	HIGH	0.04	100%	–	–	–	NA
c.781A > T	p.Ser261Cys	HIGH	0.05	95.2% ± 0.6%	–	△(E7q36) [2.0% ± 0.3%]	955-nt [2.8% ± 0.4%]	PVS1 (2%)
c.673G > C	p.Val225Leu	HIGH	0.13	100%	–	–	–	NA
c.672G > C	p.Glu224Asp	HIGH	0.98	–	▼(E6q5) [93.9% ± 0.4%] △(E6) [6.1% ± 0.4%]	–	–	PVS1 (100%)
c.672G > T	p.Glu224Asp	HIGH	0.97	–	▼(E6q5) [95.7% ± 1.7%] △(E6) [4.3% ± 1.7%]	–	–	PVS1 (100%)
c.671A > C	p.Glu224Ala	HIGH	0.77	–	▼(E6q5) [87.6% ± 0.2%] △(E6) [7.1% ± 0.1%]	–	1043-nt [5.3% ± 0.2%]	PVS1 (94.7%)
c.671A > G	p.Glu224Gly	HIGH	0.73	52.0% ± 1.0%	▼(E6q5) [23.4% ± 0.8%] △(E6) [24.6% ± 0.2%]	–	–	PVS1 (48%)
c.671A > T	p.Glu224Val	HIGH	0.85	5.9% ± 0.1%	▼(E6q5) [81.2% ± 0.3%] △(E6) [12.9% ± 0.4%]	–	–	PVS1 (94.1%)
c.560G > C	p.Gly187Ala	HIGH	0.29	37.3% ± 0.2%	△(E6) [62.7% ± 0.2%]	–	–	PVS1 (62.7%)
c.560G > T	p.Gly187Val	HIGH	0.38	41.8% ± 1.8%	△(E6) [58.2% ± 1.8%]	–	–	PVS1 (58.7%)
c.559G > A	p.Gly187Ser	HIGH	0.56	–	Δ(E5q46) [88.9 ± 2.3%]	–	417-nt [11.1% ± 2.8%]	PVS1 (88.9%)
c.559G > C	p.Gly187Arg	HIGH	0.4	–	Δ(E5q46) [100%]	–	–	PVS1 (100%)
c.559G > T	p.Gly187Cys	HIGH	0.4	21.9% ± 0.2%	Δ(E5q46) [69.8% ± 0.2%] ▼(I5) [5.2% ± 0.1%]	–	953-nt [1.2%] 1000-nt [1.9%−0.1%]	PVS1 (75%)
c.374C > G	p.Thr125Arg	HIGH	0.15	100%	–	–	–	NA

MES = MaxEntScan; PTC = Premature termination codon; SAI = SpliceAI

All variants producing aberrant transcripts met PVS1, except one producing a transcript (ΔE9p3 [100%]) for which PVS1_NA was assigned based on the BayesDel bioinformatic predictions for single amino acid deletions at positions spanning the p.Ala307Leu308delinsVal variant (as per current *TP53* VCEP specifications). Of the 19 PVS1-assigned aberrant transcript variants, 13 produced transcripts had a total expression higher than 50%, eight had a total expression higher than 80%, and three had a full expression of 100%.

### SpliceAI demonstrates highest predictive performance at currently used VCEP cutoffs

Performance for Splice and MES was evaluated against variants without splice impact (i.e., FL transcript expression at 100%) and variants with a high splice impact (PVS1-assigned aberrant transcript expression > 80%), as well as other variants with intermediate aberrant expression ranges (Table 6). Overall, of the variants not predicted to alter splicing, SpliceAI had the highest proportion of true negatives at the ≤ 0.1 cutoff (80%), in comparison to the < 0.5 cut-off (41.2%) and MES (18.2%). On the other hand, of the variants predicted to alter splicing, the proportion of true positives was not markedly different for the three approaches: 33–38% for SpliceAI prediction at either cutoff or MES when considering only variants resulting in a high splice impact of > 80% aberrant transcripts, and 55–57% when considering all variants producing an aberrant transcript with expression higher than 50%.

**Table 6:** Comparison of predictive performance of each splicing predictor category

Splicing result category*	SpliceAI < 0.5 (%)	SpliceAI ≥ 0.5 (%)		SpliceAI ≤ 0.1 (%)	SpliceAI ≥ 0.2 (%)		MES (low or none) (%)	MES (high) (%)
No splice impact (FL expression = 100%)	7 (41.2%)	5 (13.5%)		4 (80%)	5 (10.9%)		4 (18.2%)	7 (22.6%)
PVS1-assigned aberrant transcript expression < 50%	5 (29.4%)	11 (29.7%)		1 (20%)	15 (32.6%)		9 (40.9%)	7 (22.6%)
PVS1-assigned aberrant transcript expression = 50–80%	4 (23.5%)	7 (18.9%)		0	11 (23.9%)		5 (22.7%)	6 (19.3%)
High splice impact (aberrant transcript expression > 80%)	1 (5.9%)	14 (37.8%)		0	15 (32.6%)		4 (18.2%)	11 (35.5%)
Total	17	37		5	46		22	31

^*^This table excludes four variants producing only uncharacterised transcripts and one variant producing an aberrant transcript assigned with PVS1_NA at 100%, as well as variants in the intermediate 0.1–0.2 SpliceAI and “Moderate” MES categories, none of which produced any PVS1-assigned aberrant transcript

FL = Full-length, PTC = Premature termination codon

### Lack of strong correlation between intermediate SpliceAI scores and aberrant transcript expression

**Fig. 1:**
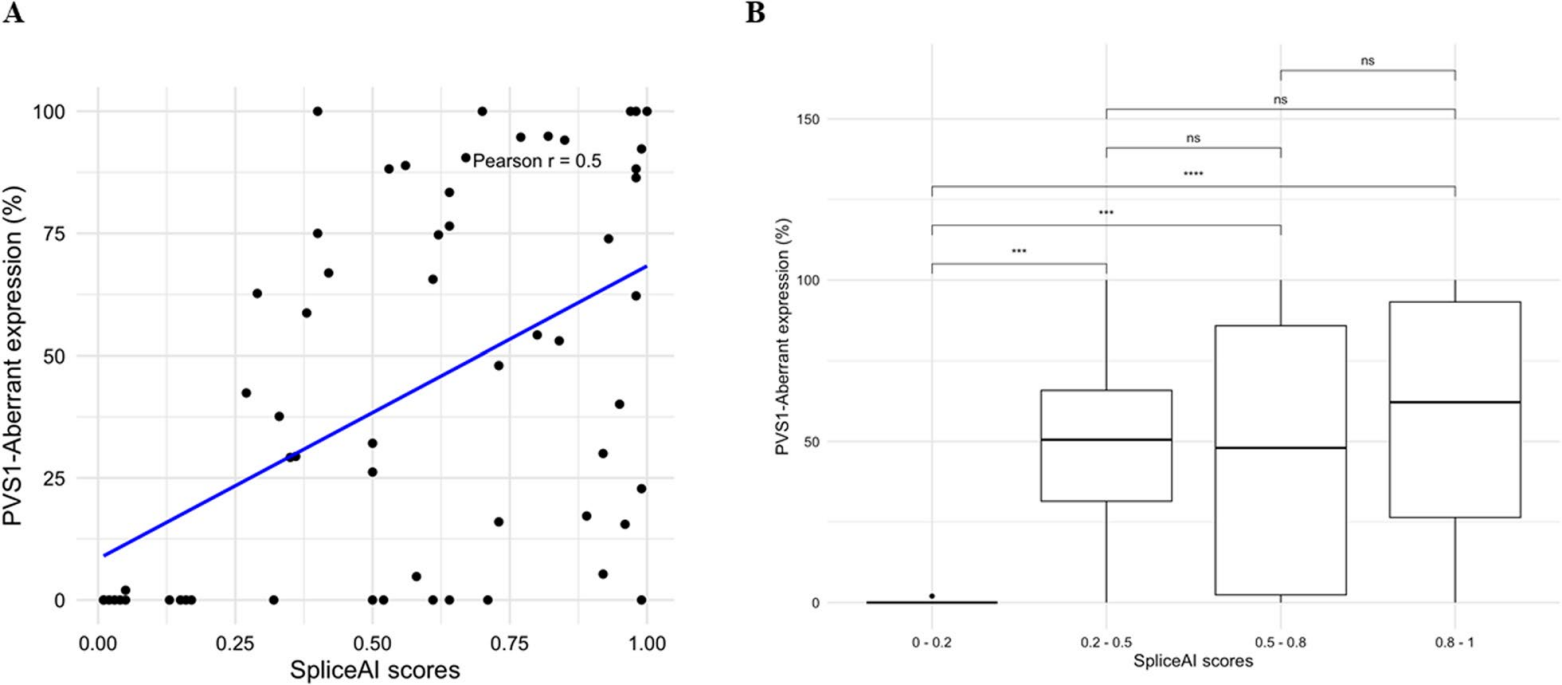
Correlation between SpliceAI maximum scores and the expression level of the corresponding PVS1-assigned aberrant transcripts, using individual scores (**A**) and score ranges (**B**)

With regards to the correlation between SpliceAI maximum delta scores and level of PVS1-assigned aberrant transcript expression, there was a positive moderate correlation of 0.50 using Pearson’s correlation analysis (Fig. 1A). Upon analysing the data using box plots for different score bins, it was evident that there was a clear distinction in the transcript expression distribution for variants with SpliceAI scores below 0.2, as most of these variants exhibited 0% expression of PVS1-assigned aberrant transcripts (Fig. 1B). In contrast, no significant differences were observed among the other three score groups, although variants with SpliceAI scores over 0.8 generally exhibited somewhat higher aberrant transcript expression.

### SpliceAI-10k calculator is effective at predicting specific aberrant transcripts

Specific types of splicing aberrations were predicted using the SAI-10k-calc algorithm, which interprets the combination of all SpliceAI delta scores and delta positions. Results showed that SAI-10k-calc can predict specific aberrant transcripts with at least 3% of overall expression (Supplementary Table 3). Splicing aberrations matched for all positive control variants for at least one of the transcripts, except for one variant (c.782 + 1G > A), in which SpliceAI predicted the loss of donor site but not the loss of its acceptor site pair, thus Δ(E7) observed in the assay was not predicted.

**Fig. 2:**
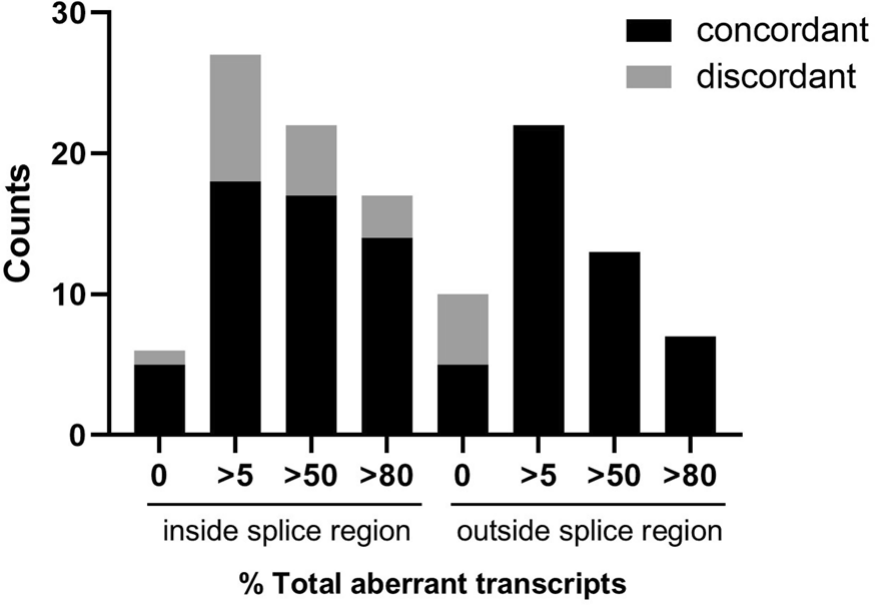
Predictive performance of SpliceAI-10k calculator inside or outside the splice region and at different percentage aberrant transcript cutoffs. Data presented for variants inside the splice region includes the splice site dinucleotide variants. A variant having a predicted aberrant transcript that matches with at least one variant-induced transcript in the assay is counted as a concordant observation

We compared the predicted transcripts against the observed assay results at different levels (> 5%, > 50%, and > 80%) of total characterised aberrant transcripts for control and test variants (Fig. 2, Supplementary Table 3). Of the 49 variants with > 5% aberrant transcript expression, 40 (81.6%) had at least one predicted transcript that matched with assay results. All nine variants with observed splicing impact but no predicted aberration were located inside the splice region. Variants inside the splice region, including those located at the splice site dinucleotides, often alter splicing through loss of donor/acceptor site. SpliceAI correctly predicted the loss of donor site for six variants inside the donor splice region, but did not accurately predict the precise location of the loss of their acceptor site pair, resulting in a lower number of matched transcripts with exon skipping or intron retention. Transcript prediction was better for variants located outside of the splice region at all three levels of aberrant transcript expression compared to variants inside the splice region. Outside the splice region, all variants with > 5% aberrant transcript expression had matching predicted aberrant transcript(s). Therefore, the SAI-10k-calc default settings had high sensitivity for exonic variants outside the splice region that lead to usage of new or cryptic splice sites, generating transcripts with partial exon deletion or partial intron retention.

Of the 16 test variants that did not induce any characterised aberrant transcript, 10 (62.5%) with SpliceAI maximum delta scores ranging from 0.01 to 0.17, were correctly predicted as having no splicing impact (Fig. 1, Supplementary Table 3). Six of the 16 variants located in exon 4 or 8, with SpliceAI maximum delta scores ranging from 0.5 to 0.71, were incorrectly predicted to generate transcripts with partial exon deletion. Further inspection using SpliceAI-visual [21] revealed that exons 4 and 8 are bounded by strong native splice sites with SpliceAI reference scores > 0.97. At any predicted splice site position, SpliceAI provides the alternate score, which reflects the splice site strength after introducing a variant into the reference sequence. The six false positive variants had SpliceAI alternate scores for donor/acceptor gain ranging from 0.38 to 0.72 that were weaker than the alternate scores for the native splice sites (Supplementary Fig. 3). This means that the false positive predictions were due to failure of the default algorithm to consider the splice site competition condition (i.e., strength of native vs new splice site).

However, the splice site competition condition was not applicable to certain prioritised variants. The SAI-10k-calc default algorithm correctly predicted the transcripts with partial exon deletion for nine variants although the alternate scores for the new donor/acceptor were lower by more than 0.2 compared to the native splice sites (Supplementary Table 4). For example, c.559G > T led to donor gain with an alternate score of 0.41, a considerably weaker score than the native donor (0.97), but usage of the new donor site was still observed in the assay.

### Splicing assay results can contribute to *TP53* variant classification

Focusing on variants with a very strong splicing impact (PVS1 (RNA)_Variable weight expression higher than 80%), addition of publicly available data indicates that at least 14 of the test variants could be classified as P/LP using *TP53* specifications v2 (Table 7). All these variants had SpliceAI scores ≥ 0.4, all were absent from the FLOSSIES database and four were observed in cancer probands in the *TP53* database. Only one of these variants (c.318C > G (p.Ser106Arg)) was already classified as LP in ClinVar, with a single submission from Invitae which mentioned a positive splicing prediction. One variant, c.314G > T (p.Gly105Val), currently classified in ClinVar as LP by a single submitter with no evidence summary provided, and in the absence of privately-held clinical data available, stayed as VUS in this study with 5 points (PVS1_Strong and PM2_Supporting codes applied).

**Table 7:** *TP53* variants classified as pathogenic or benign after incorporating splicing assay results

Variant	ClinVar (as at October 2024)	*TP53*-ACMG/AMP v2 codes applied (suggested class)
*Variants classified as P/LP using PVS1 (RNA)*		
c.782G > C (p.Ser261Thr)	Uncertain significance (criteria provided, single submitter)	PVS1 (RNA) (83.4%) and PM2_Supporting (LP)
c.782G > T (p.Ser261Ile)	–	PVS1 (RNA) (88.2%) and PM2_Supporting (LP)
c.672G > C (p.Glu224Asp)	Uncertain significance (criteria provided, single submitter)	PVS1 (RNA) (100%) and PM2_Supporting (LP)
c.672G > T (p.Glu224Asp)	Uncertain significance (criteria provided, single submitter)	PVS1 (RNA) (100%) and PM2_Supporting (LP)
c.671A > C (p.Glu224Ala)	Uncertain significance (criteria provided, multiple submitters, no conflicts)	PVS1 (RNA) (94.7%) and PM2_Supporting (LP)
c.671A > T (p.Glu224Val)	–	PVS1 (RNA) (94.1%) and PM2_Supporting (LP)
c.559G > A (p.Gly187Ser)	Uncertain significance (criteria provided, multiple submitters, no conflicts)	PVS1 (RNA) (88.9%) and PM2_Supporting (LP)
c.559G > C (p.Gly187Arg)	Conflicting classifications of pathogenicity (1xLP, 1xVUS)	PVS1 (RNA) (100%) and PM2_Supporting (LP)
c.410T > A (p.Leu137Gln)	–	PVS1 (RNA) (100%) and PM2_Supporting (LP)
c.356C > G (p.Ala119Gly)	Conflicting classifications of pathogenicity (1xLP, 1xVUS)	PVS1 (RNA) (94.9%), PM2_Supporting and PS4_Supporting (P)
c.318C > G (p.Ser106Arg)	Likely pathogenic (criteria provided, single submitter)	PVS1 (RNA) (100%) and PM2_Supporting (LP)
c.182A > G (p.Asp61Gly)	Uncertain significance (criteria provided, multiple submitters, no conflicts)	PVS1 (RNA) (88.2%), PM2_Supporting and PP4 (P)
c.178C > A (p.Pro60Thr)	–	PVS1 (RNA) (90.5%) and PM2_Supporting (LP)
c.50A > T (p.Glu17Val)	–	PVS1 (RNA) (92.3%) and PM2_Supporting (LP)
*Missense variants classified as P/LP after ruling out splicing*		
c.817C > A (p.Arg273Ser)	Pathogenic (criteria provided, multiple submitters, no conflicts)	PM2_Supporting, PS4_Supporting, PS3, PP3_Moderate, PM1 (P)
c.811G > A (p.Glu271Lys)	Conflicting classifications of pathogenicity (3xLP, 2xVUS)	PM2_Supporting, PS3_Moderate, PP3, PM1, PP4 (LP)
c.797G > T (p.Gly266Val)	Conflicting classifications of pathogenicity (1xP, 3xVUS)	PM2_Supporting, PS3, PP3_Moderate, PM1 (LP)
c.374C > G (p.Thr125Arg)	Pathogenic/Likely pathogenic (criteria provided, multiple submitters, no conflicts)	PM2_Supporting, PS4_Supporting, PS3, PP3_Moderate, PM1_Supporting (LP)
c.325T > A (p.Phe109Ile)	Likely pathogenic (criteria provided, multiple submitters, no conflicts)	PM2_Supporting, PS4_Supporting (data from ClinVar Accession SCV004098979.1), PS3 (LP)
*Missense variants classified as LB after ruling out splicing*		
c.919G > T (p.Ala307Ser)	–	PM2_Supporting, BS3, BP4_Moderate (LB)
c.781A > C (p.Ser261Arg)	–	PM2_Supporting, BS3, BP4 (LB)
c.781A > G (p.Ser261Gly)	Conflicting classifications of pathogenicity (1xLB, 1xVUS)	PM2_Supporting, BS3, BP4_Moderate (LB)
c.673G > C (p.Val225Leu)	–	PM2_Supporting, BS3, BP4_Moderate (LB)
*Synonymous variants classified as LB after ruling out splicing*		
c.837G > C (p.Gly279 =)	–	PM2_Supporting, BP7_Strong (RNA) (LB)
c.837G > T (p.Gly279 =)	–	PM2_Supporting, BP7_Strong (RNA) (LB)
c.816G > A (p.Val272 =)	Likely benign (criteria provided, single submitter with no evidence summary)	PM2_Supporting, BP7_Strong (RNA) (LB)
c.114A > G (p.Gln38 =)	–	PM2_Supporting, BP7_Strong (RNA) (LB)

LB = Likely benign, LP = Likely pathogenic, P = Pathogenic, VUS = Variant of uncertain significance

Similarly, we found that experimental evidence of no splicing impact could contribute to variant classification by allowing use of the relevant functional and computational codes. Specifically, of the 16 variants predicted to alter splicing by either SpliceAI or MES but which did not produce any aberrant transcript according to our results, 13 could now be classified using *TP53* specifications v2: five missense variants as P/LP, four missense variants as LB, and four synonymous variants as LB. This evaluation would add nine new clinically-relevant classifications.

## Discussion

This study provides new evidence regarding the disease mechanism of specific *TP53* variants, highlighting their splicing effects, especially for underexplored variants such as apparent synonymous variants outside splice regions and missense variants.

Our results support the use of SpliceAI at the ≤ 0.1 and ≥ 0.2 cutoffs, determined by calibration using other genes [2], as an effective approach for *TP53* germline classification, especially to identify true negatives. A key question for future research is whether we can infer level of splice impact, and by extension, pathogenicity, from the range of SpliceAI maximum delta scores. All positive controls scored above 0.98, and all but one had PVS1-assigned aberrant transcript expression levels exceeding 92.6%. For the remaining variants that were all located outside of the splice site dinucleotide positions, we observed an apparent moderate positive correlation between SpliceAI scores and the expression levels of PVS1-assigned aberrant transcripts. However, this correlation was substantially influenced by a cluster of variants with SpliceAI scores below 0.2 with 0% aberrant expression, and we found no significant differences in expression levels among variants with SpliceAI scores ≥ 0.2. Larger studies will be valuable for further exploring the relationship between SpliceAI scores and aberrant transcript expression. In contrast, predictions of expected aberrant transcripts using the SpliceAI-10k calculator [16] were generally concordant with the observed assay results. Forty of the 49 variants that impact splicing with > 5% total aberrant transcript expression had predicted transcripts that were concordant with at least one transcript observed in the assay. Competition between the strength of the native splice site and the new/cryptic splice site activated by a variant, as indicated by their SpliceAI alternate scores, could explain the false positive aberrant transcript predictions. We note that SpliceAI v1.3.1 or SpliceAI Lookup (https://spliceailookup.broadinstitute.org/) does not generate the alternate score for the native splice sites in some cases, which in turn limits the ability of SAI-10k-calc to accurately predict partial exon deletion or partial intron retention. In such cases, including the six false positives observed in this study, SpliceAI-visual can inform the precise interpretation of SpliceAI scores. Further, SpliceAI predictions also revealed a potential modification of expression levels of alternative transcripts containing exons 9β or 9γ that were not captured by the minigene construct design. These findings demonstrate how additional bioinformatic prioritisation may be helpful to select likely loss of function variants to assay when resources are limited.

For *TP53*, it is still not clearly known what level of aberrant pathogenic transcript expression with different molecular mechanisms is associated with expression of disease. For the *BRCA1* and *BRCA2* tumor suppressor genes, a 20% wild-type expression of the full-length transcript has been designated as sufficient to prevent tumorigenesis by current VCEP specifications [15]. Of our positive controls, NM_000546.6(TP53):c.783-1G > A had the lowest expression of transcripts assigned a PVS1 (RNA) code (49.8%), with the remaining transcript produced predicted to be functional (△(E8p24) encoding p.Ser261_Gly262insSer). Assuming the protein coded by △(E8p24) is functional, and noting that this variant has been observed in Li-Fraumeni syndrome individuals according to multiple ClinVar submissions, it is reasonable to assume that an aberrant transcript expression of at least ~ 50% might be enough to negatively affect *TP53* expression and p53 function and predispose to tumorigenesis. For missense variants where the expression of the PVS1-assigned aberrant transcript is less than the established cutpoint, there is rationale to assign variant impact based on the combination of splicing transcript and functional data that measures missense impact only. In this study, none of the 22 missense variants with aberrant transcripts meeting PVS1 (RNA)_Variable weight at < 80% expression met PS3 at any weight based on protein-level functional data.

Our study demonstrates how RNA-based splicing data can help classify *TP53* germline variants. Using an 80% threshold for aberrant transcript expression, we classified 14 variants as pathogenic based on their high splice impact. Another 13 variants, with no splicing effect observed despite bioinformatics predictions, were classified as either pathogenic or benign following the relevant missense and synonymous routes. These updated classifications could aid in more informed medical management for patients carrying these *TP53* variants.

## Conclusions

In conclusion, this work sets the stage for improved understanding and classification of *TP53* variants using splicing predictions and assays, ultimately enhancing patient access to personalised clinical management strategies. Our study highlights that a significant number of *TP53* variants impact splicing, which underscores the necessity of thoroughly evaluating the potential impact of all variants at the nucleotide level on mRNA splicing, in addition to protein level bioinformatic prediction of missense substitution, the two-step process recommended by the *TP53* VCEP. While further research is needed to investigate potential correlation between maximum SpliceAI scores and the actual levels of aberrant splicing induced by a given variant, data show that the SpliceAI-10k calculator can provide valuable insight into the types of aberrant transcripts expected to be generated by variants that impact splicing. Data from this study also supports future investigation of a *TP53*-specific threshold of 50% aberration from the variant allele as appropriate for assigning mRNA-related evidence towards variant pathogenicity. Importantly, the mRNA data generated from this study has already facilitated the suggested classification of 27 out of 59 variants, and all findings are now available for variant curation efforts.

## Supplementary Information

Supplementary Figure 1.

Supplementary Figure 2.

Supplementary Figure 3.

Supplementary Tables 1–4.

## Data Availability

All data generated or analysed during this study are included in this published article and its supplementary information files.

## Abbreviations

ALT: Alternate score
AG: Acceptor gain
AL: Acceptor loss
B: Benign
DG: Donor gain
DL: Donor loss
FL: Full-length
LB: Likely benign
LP: Likely pathogenic
NA: Not applicable
P: Pathogenic
PTC: Premature termination codon
RT-PCR: Reverse transcription polymerase chain reaction
SAI: SpliceAI
SVI: Sequence Variant Interpretation
VCEP: Variant Curation Expert Panel
VUS: Variant of uncertain significance
