# Supplementary material for: Exploring the role of splicing in TP53 variant pathogenicity through predictions and minigene assays
Source: Hum Genomics. 2025 Jan 8;19:2. doi: 10.1186/s40246-024-00714-5 (PMC11715486; doi:10.1186/s40246-024-00714-5)
Supplement: Supplementary file 1 — Supplementary Figure 1. [file 40246_2024_714_MOESM1_ESM.pptx]

## Slide 1
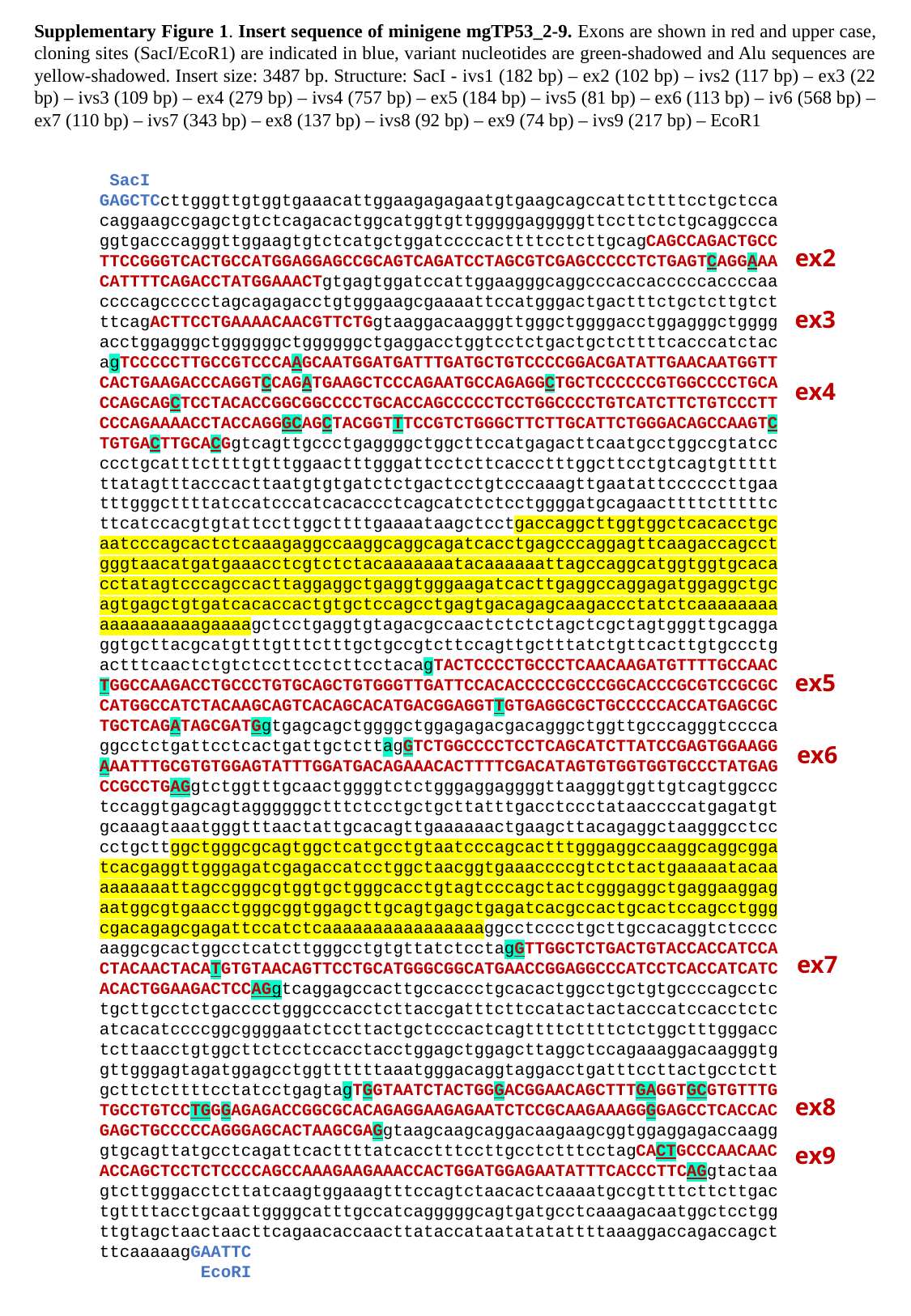

Supplementary Figure 1. Insert sequence of minigene mgTP53_2-9. Exons are shown in red and upper case, cloning sites (SacI/EcoR1) are indicated in blue, variant nucleotides are green-shadowed and Alu sequences are yellow-shadowed. Insert size: 3487 bp. Structure: SacI - ivs1 (182 bp) – ex2 (102 bp) – ivs2 (117 bp) – ex3 (22 bp) – ivs3 (109 bp) – ex4 (279 bp) – ivs4 (757 bp) – ex5 (184 bp) – ivs5 (81 bp) – ex6 (113 bp) – iv6 (568 bp) – ex7 (110 bp) – ivs7 (343 bp) – ex8 (137 bp) – ivs8 (92 bp) – ex9 (74 bp) – ivs9 (217 bp) – EcoR1
 SacI
GAGCTCcttgggttgtggtgaaacattggaagagagaatgtgaagcagccattcttttcctgctccacaggaagccgagctgtctcagacactggcatggtgttgggggagggggttccttctctgcaggcccaggtgacccagggttggaagtgtctcatgctggatccccacttttcctcttgcagCAGCCAGACTGCCTTCCGGGTCACTGCCATGGAGGAGCCGCAGTCAGATCCTAGCGTCGAGCCCCCTCTGAGTCAGGAAACATTTTCAGACCTATGGAAACTgtgagtggatccattggaagggcaggcccaccacccccaccccaaccccagccccctagcagagacctgtgggaagcgaaaattccatgggactgactttctgctcttgtctttcagACTTCCTGAAAACAACGTTCTGgtaaggacaagggttgggctggggacctggagggctggggacctggagggctggggggctggggggctgaggacctggtcctctgactgctcttttcacccatctacagTCCCCCTTGCCGTCCCAAGCAATGGATGATTTGATGCTGTCCCCGGACGATATTGAACAATGGTTCACTGAAGACCCAGGTCCAGATGAAGCTCCCAGAATGCCAGAGGCTGCTCCCCCCGTGGCCCCTGCACCAGCAGCTCCTACACCGGCGGCCCCTGCACCAGCCCCCTCCTGGCCCCTGTCATCTTCTGTCCCTTCCCAGAAAACCTACCAGGGCAGCTACGGTTTCCGTCTGGGCTTCTTGCATTCTGGGACAGCCAAGTCTGTGACTTGCACGgtcagttgccctgaggggctggcttccatgagacttcaatgcctggccgtatccccctgcatttcttttgtttggaactttgggattcctcttcaccctttggcttcctgtcagtgtttttttatagtttacccacttaatgtgtgatctctgactcctgtcccaaagttgaatattccccccttgaatttgggcttttatccatcccatcacaccctcagcatctctcctggggatgcagaacttttctttttcttcatccacgtgtattccttggcttttgaaaataagctcctgaccaggcttggtggctcacacctgcaatcccagcactctcaaagaggccaaggcaggcagatcacctgagcccaggagttcaagaccagcctgggtaacatgatgaaacctcgtctctacaaaaaaatacaaaaaattagccaggcatggtggtgcacacctatagtcccagccacttaggaggctgaggtgggaagatcacttgaggccaggagatggaggctgcagtgagctgtgatcacaccactgtgctccagcctgagtgacagagcaagaccctatctcaaaaaaaaaaaaaaaaaagaaaagctcctgaggtgtagacgccaactctctctagctcgctagtgggttgcaggaggtgcttacgcatgtttgtttctttgctgccgtcttccagttgctttatctgttcacttgtgccctgactttcaactctgtctccttcctcttcctacagTACTCCCCTGCCCTCAACAAGATGTTTTGCCAACTGGCCAAGACCTGCCCTGTGCAGCTGTGGGTTGATTCCACACCCCCGCCCGGCACCCGCGTCCGCGCCATGGCCATCTACAAGCAGTCACAGCACATGACGGAGGTTGTGAGGCGCTGCCCCCACCATGAGCGCTGCTCAGATAGCGATGgtgagcagctggggctggagagacgacagggctggttgcccagggtccccaggcctctgattcctcactgattgctcttagGTCTGGCCCCTCCTCAGCATCTTATCCGAGTGGAAGGAAATTTGCGTGTGGAGTATTTGGATGACAGAAACACTTTTCGACATAGTGTGGTGGTGCCCTATGAGCCGCCTGAGgtctggtttgcaactggggtctctgggaggaggggttaagggtggttgtcagtggccctccaggtgagcagtaggggggctttctcctgctgcttatttgacctccctataaccccatgagatgtgcaaagtaaatgggtttaactattgcacagttgaaaaaactgaagcttacagaggctaagggcctcccctgcttggctgggcgcagtggctcatgcctgtaatcccagcactttgggaggccaaggcaggcggatcacgaggttgggagatcgagaccatcctggctaacggtgaaaccccgtctctactgaaaaatacaaaaaaaaattagccgggcgtggtgctgggcacctgtagtcccagctactcgggaggctgaggaaggagaatggcgtgaacctgggcggtggagcttgcagtgagctgagatcacgccactgcactccagcctgggcgacagagcgagattccatctcaaaaaaaaaaaaaaaaggcctcccctgcttgccacaggtctccccaaggcgcactggcctcatcttgggcctgtgttatctcctagGTTGGCTCTGACTGTACCACCATCCACTACAACTACATGTGTAACAGTTCCTGCATGGGCGGCATGAACCGGAGGCCCATCCTCACCATCATCACACTGGAAGACTCCAGgtcaggagccacttgccaccctgcacactggcctgctgtgccccagcctctgcttgcctctgacccctgggcccacctcttaccgatttcttccatactactacccatccacctctcatcacatccccggcggggaatctccttactgctcccactcagttttcttttctctggctttgggacctcttaacctgtggcttctcctccacctacctggagctggagcttaggctccagaaaggacaagggtggttgggagtagatggagcctggttttttaaatgggacaggtaggacctgatttccttactgcctcttgcttctcttttcctatcctgagtagTGGTAATCTACTGGGACGGAACAGCTTTGAGGTGCGTGTTTGTGCCTGTCCTGGGAGAGACCGGCGCACAGAGGAAGAGAATCTCCGCAAGAAAGGGGAGCCTCACCACGAGCTGCCCCCAGGGAGCACTAAGCGAGgtaagcaagcaggacaagaagcggtggaggagaccaagggtgcagttatgcctcagattcacttttatcacctttccttgcctctttcctagCACTGCCCAACAACACCAGCTCCTCTCCCCAGCCAAAGAAGAAACCACTGGATGGAGAATATTTCACCCTTCAGgtactaagtcttgggacctcttatcaagtggaaagtttccagtctaacactcaaaatgccgttttcttcttgactgttttacctgcaattggggcatttgccatcagggggcagtgatgcctcaaagacaatggctcctggttgtagctaactaacttcagaacaccaacttataccataatatatattttaaaggaccagaccagctttcaaaaagGAATTC
 EcoRI
ex2
ex3
ex4
ex5
ex6
ex7
ex8
ex9
