# Supplementary material for: Exploring the role of splicing in TP53 variant pathogenicity through predictions and minigene assays
Source: Hum Genomics. 2025 Jan 8;19:2. doi: 10.1186/s40246-024-00714-5 (PMC11715486; doi:10.1186/s40246-024-00714-5)
Supplement: Supplementary file 2 — Supplementary Figure 2. [file 40246_2024_714_MOESM2_ESM.pptx]

## Slide 1
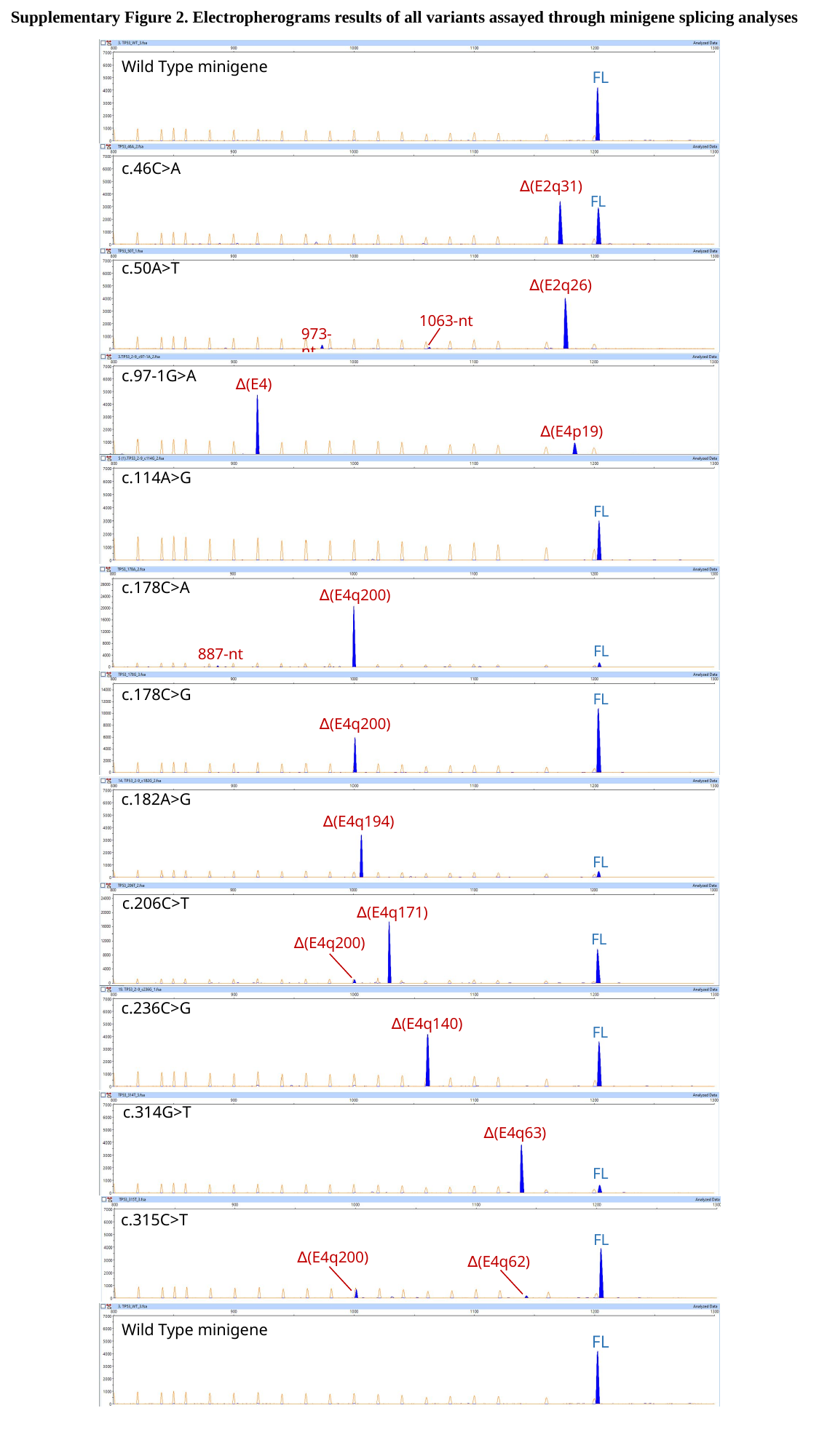

Supplementary Figure 2. Electropherograms results of all variants assayed through minigene splicing analyses
Wild Type minigene
FL
c.46C>A
∆(E2q31)
FL
c.50A>T
∆(E2q26)
1063-nt
973-nt
c.97-1G>A
∆(E4)
∆(E4p19)
c.114A>G
FL
c.178C>A
∆(E4q200)
FL
887-nt
c.178C>G
FL
∆(E4q200)
c.182A>G
∆(E4q194)
FL
c.206C>T
∆(E4q171)
FL
∆(E4q200)
c.236C>G
∆(E4q140)
FL
c.314G>T
∆(E4q63)
FL
c.315C>T
FL
∆(E4q200)
∆(E4q62)
Wild Type minigene
FL

## Slide 2
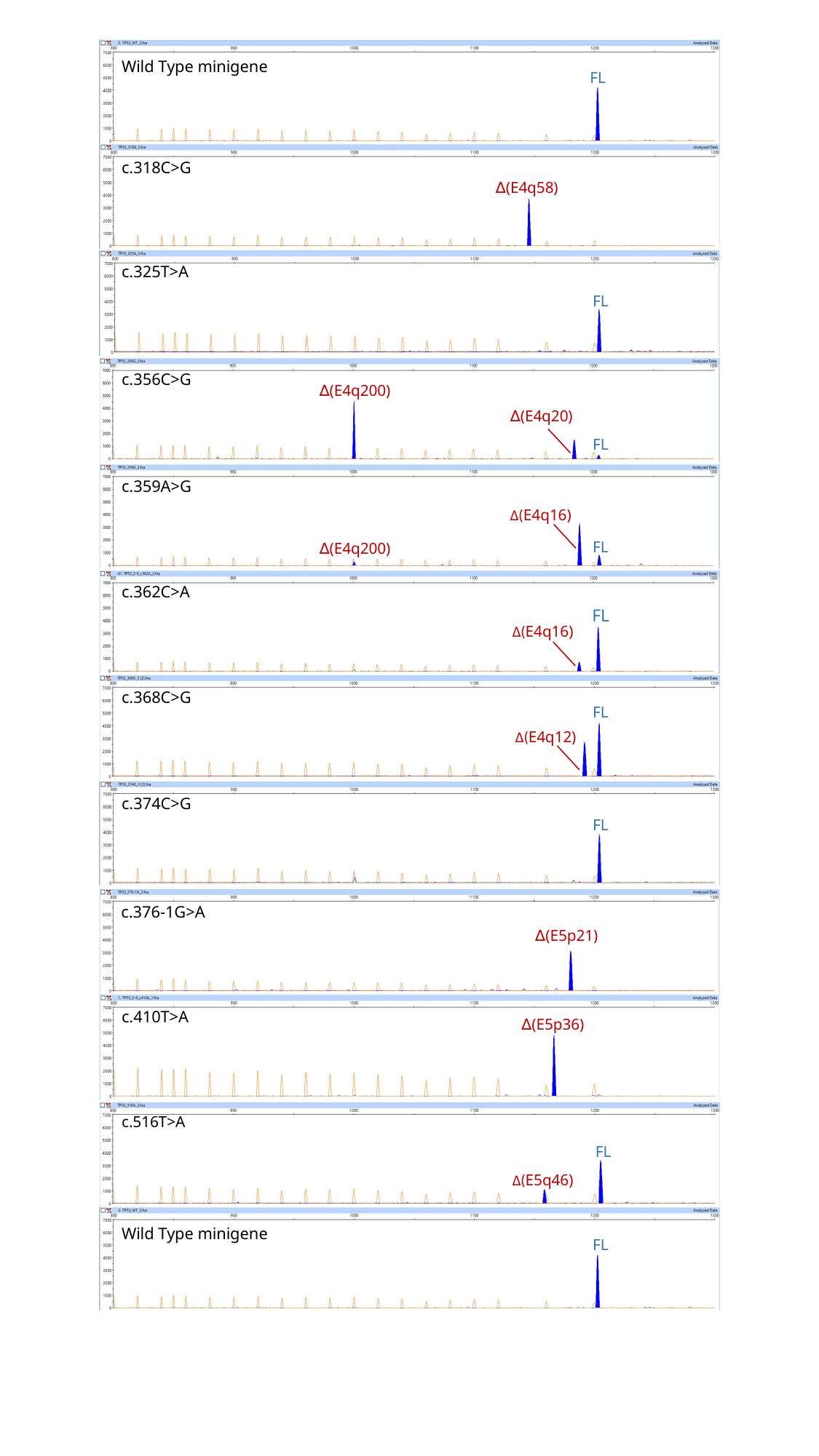

Wild Type minigene
FL
c.318C>G
∆(E4q58)
c.325T>A
FL
c.356C>G
∆(E4q200)
∆(E4q20)
FL
c.359A>G
Δ(E4q16)
FL
∆(E4q200)
c.362C>A
FL
Δ(E4q16)
c.368C>G
FL
Δ(E4q12)
c.374C>G
FL
c.376-1G>A
∆(E5p21)
c.410T>A
∆(E5p36)
c.516T>A
FL
Δ(E5q46)
Wild Type minigene
FL

## Slide 3
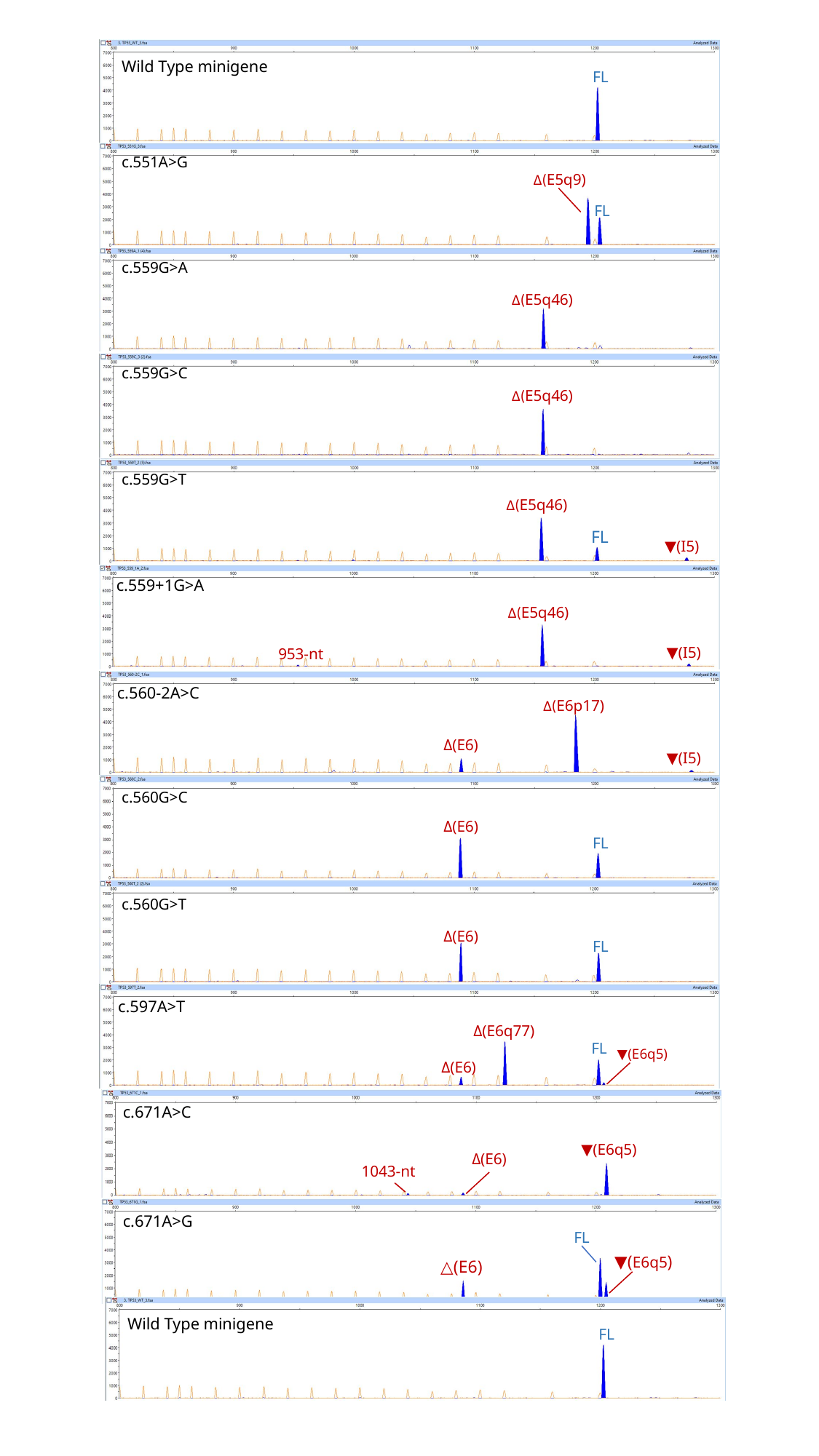

Wild Type minigene
FL
c.551A>G
Δ(E5q9)
FL
c.559G>A
Δ(E5q46)
c.559G>C
Δ(E5q46)
c.559G>T
Δ(E5q46)
FL
▼(I5)
c.559+1G>A
Δ(E5q46)
▼(I5)
953-nt
c.560-2A>C
Δ(E6p17)
Δ(E6)
▼(I5)
c.560G>C
Δ(E6)
FL
c.560G>T
Δ(E6)
FL
c.597A>T
Δ(E6q77)
FL
▼(E6q5)
Δ(E6)
c.671A>C
▼(E6q5)
Δ(E6)
1043-nt
c.671A>G
FL
▼(E6q5)
△(E6)
Wild Type minigene
FL

## Slide 4
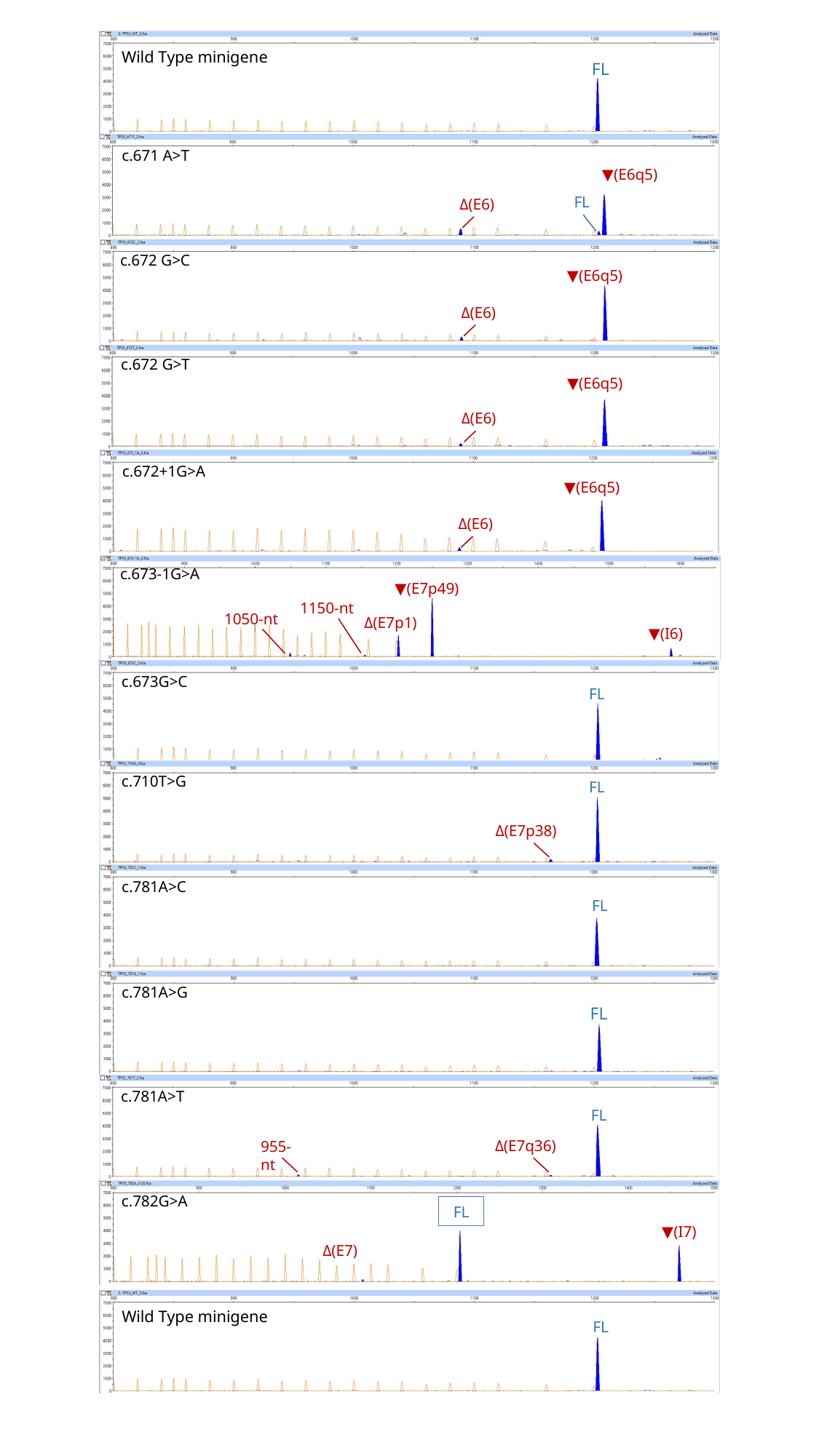

Wild Type minigene
FL
c.671 A>T
 ▼(E6q5)
FL
Δ(E6)
c.672 G>C
 ▼(E6q5)
Δ(E6)
c.672 G>T
 ▼(E6q5)
Δ(E6)
c.672+1G>A
 ▼(E6q5)
Δ(E6)
c.673-1G>A
 ▼(E7p49)
1150-nt
1050-nt
Δ(E7p1)
 ▼(I6)
c.673G>C
FL
c.710T>G
FL
Δ(E7p38)
c.781A>C
FL
c.781A>G
FL
c.781A>T
FL
Δ(E7q36)
955-nt
c.782G>A
FL
 ▼(I7)
Δ(E7)
Wild Type minigene
FL

## Slide 5
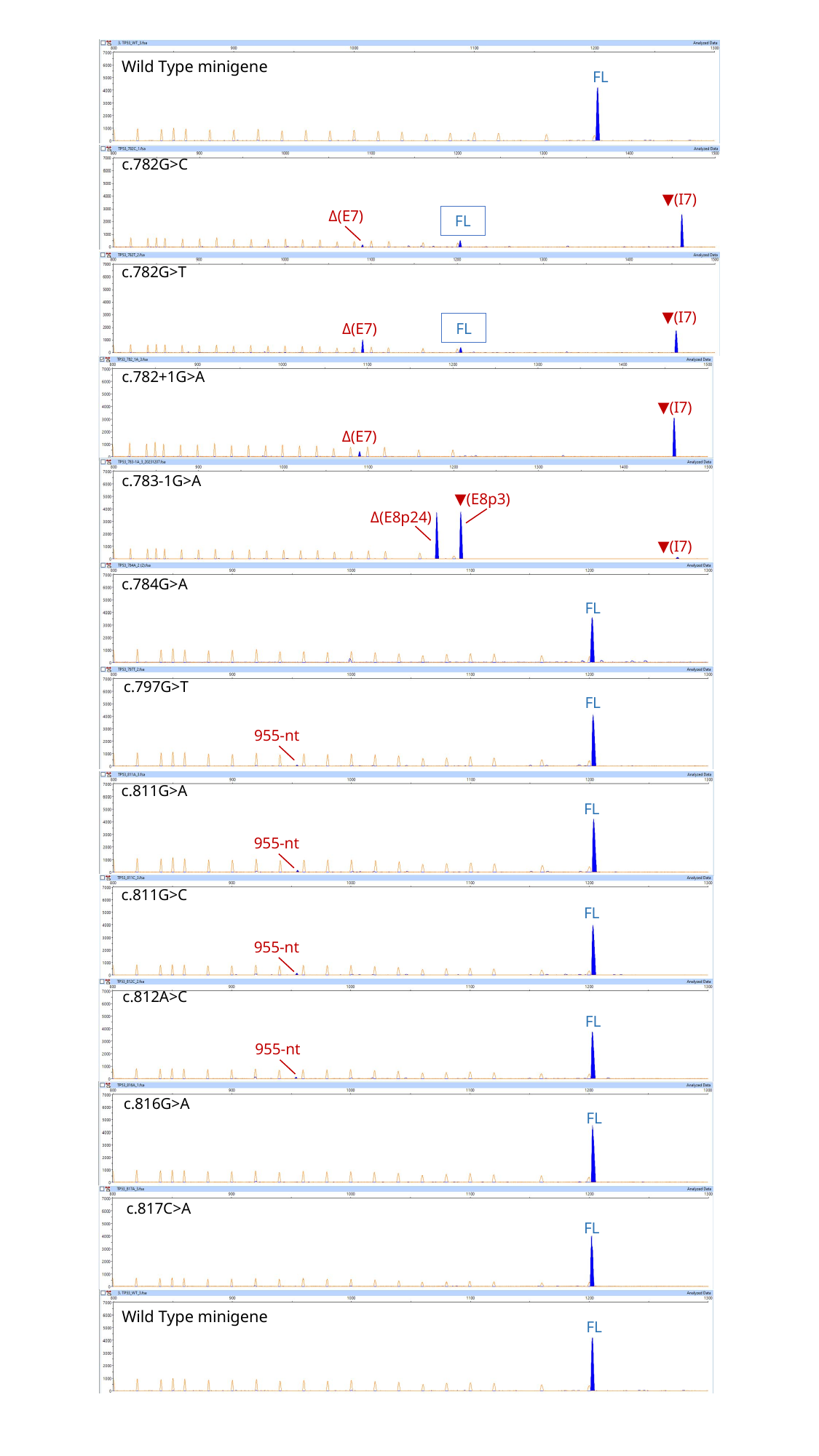

Wild Type minigene
FL
c.782G>C
 ▼(I7)
Δ(E7)
FL
c.782G>T
 ▼(I7)
FL
Δ(E7)
c.782+1G>A
 ▼(I7)
Δ(E7)
c.783-1G>A
 ▼(E8p3)
Δ(E8p24)
 ▼(I7)
c.784G>A
FL
c.797G>T
FL
955-nt
c.811G>A
FL
955-nt
c.811G>C
FL
955-nt
c.812A>C
FL
955-nt
c.816G>A
FL
c.817C>A
FL
Wild Type minigene
FL

## Slide 6
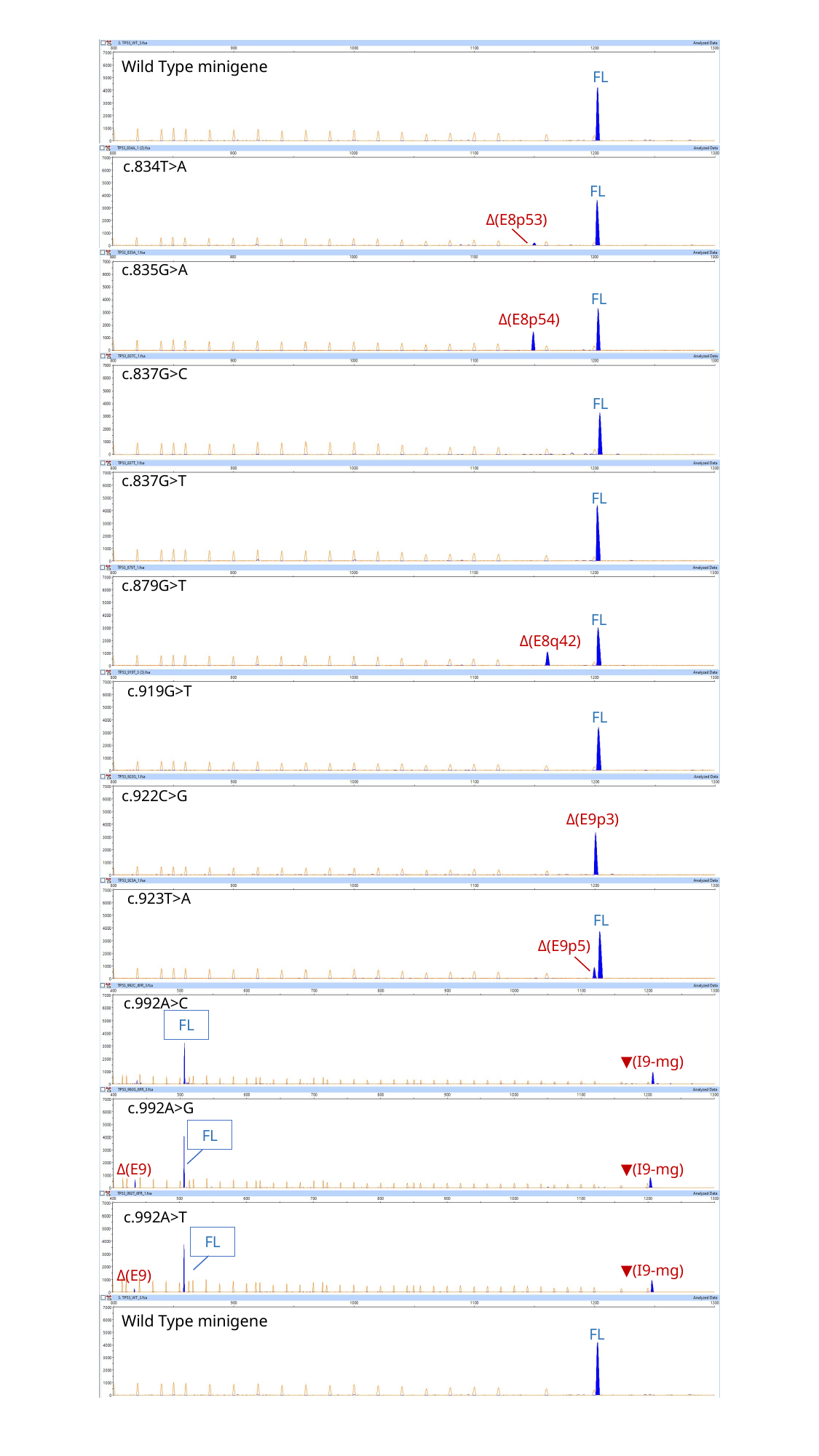

Wild Type minigene
FL
c.834T>A
FL
Δ(E8p53)
c.835G>A
FL
Δ(E8p54)
c.837G>C
FL
c.837G>T
FL
c.879G>T
FL
Δ(E8q42)
c.919G>T
FL
c.922C>G
Δ(E9p3)
c.923T>A
FL
Δ(E9p5)
c.992A>C
FL
▼(I9-mg)
c.992A>G
FL
Δ(E9)
▼(I9-mg)
c.992A>T
FL
▼(I9-mg)
Δ(E9)
Wild Type minigene
FL

## Slide 7
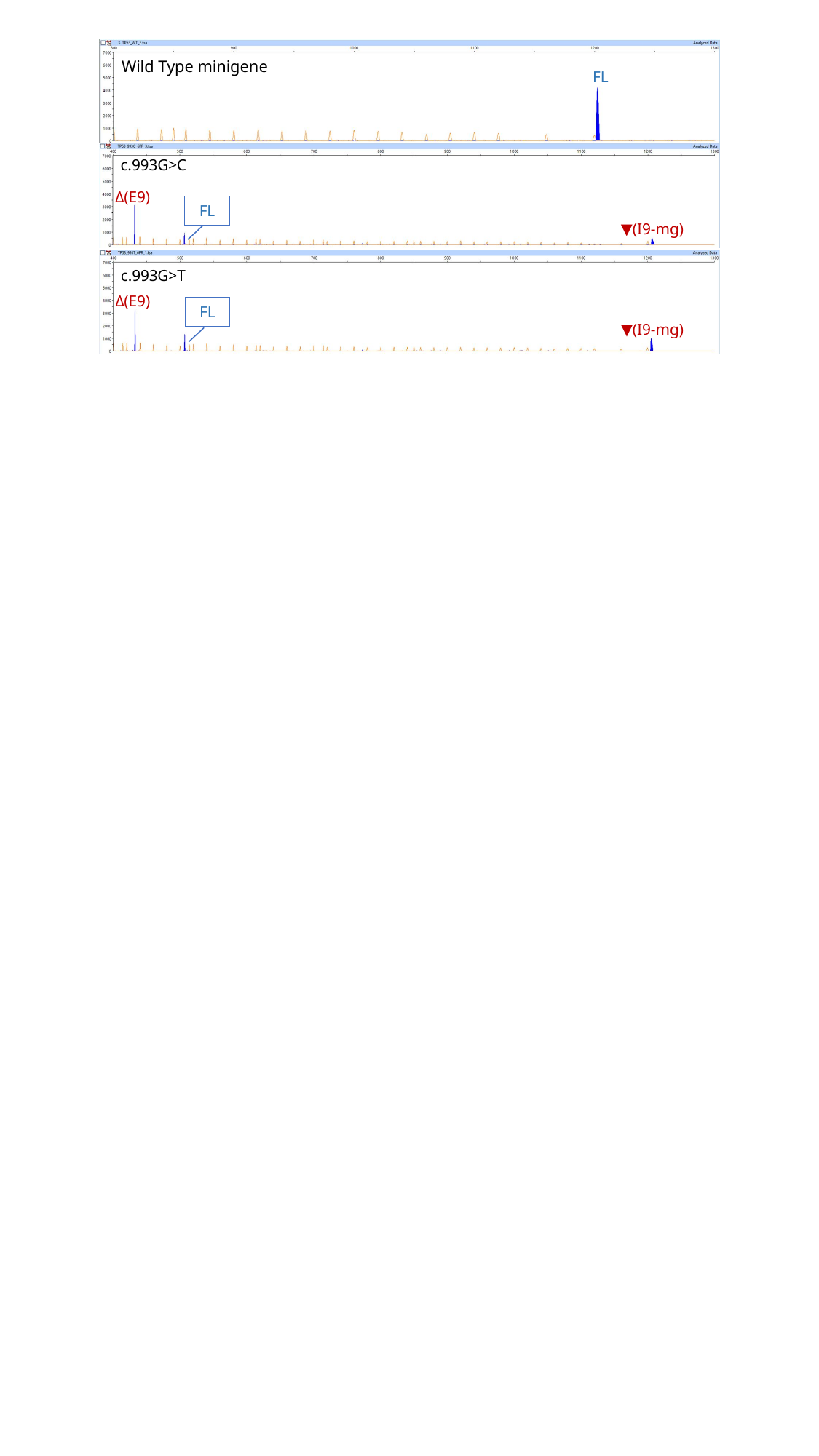

Wild Type minigene
FL
c.993G>C
Δ(E9)
FL
▼(I9-mg)
c.993G>T
Δ(E9)
FL
▼(I9-mg)
