# Supplementary material for: Exploring the role of splicing in TP53 variant pathogenicity through predictions and minigene assays
Source: Hum Genomics. 2025 Jan 8;19:2. doi: 10.1186/s40246-024-00714-5 (PMC11715486; doi:10.1186/s40246-024-00714-5)
Supplement: Supplementary file 3 — Supplementary Figure 3. [file 40246_2024_714_MOESM3_ESM.pptx]

## Slide 1
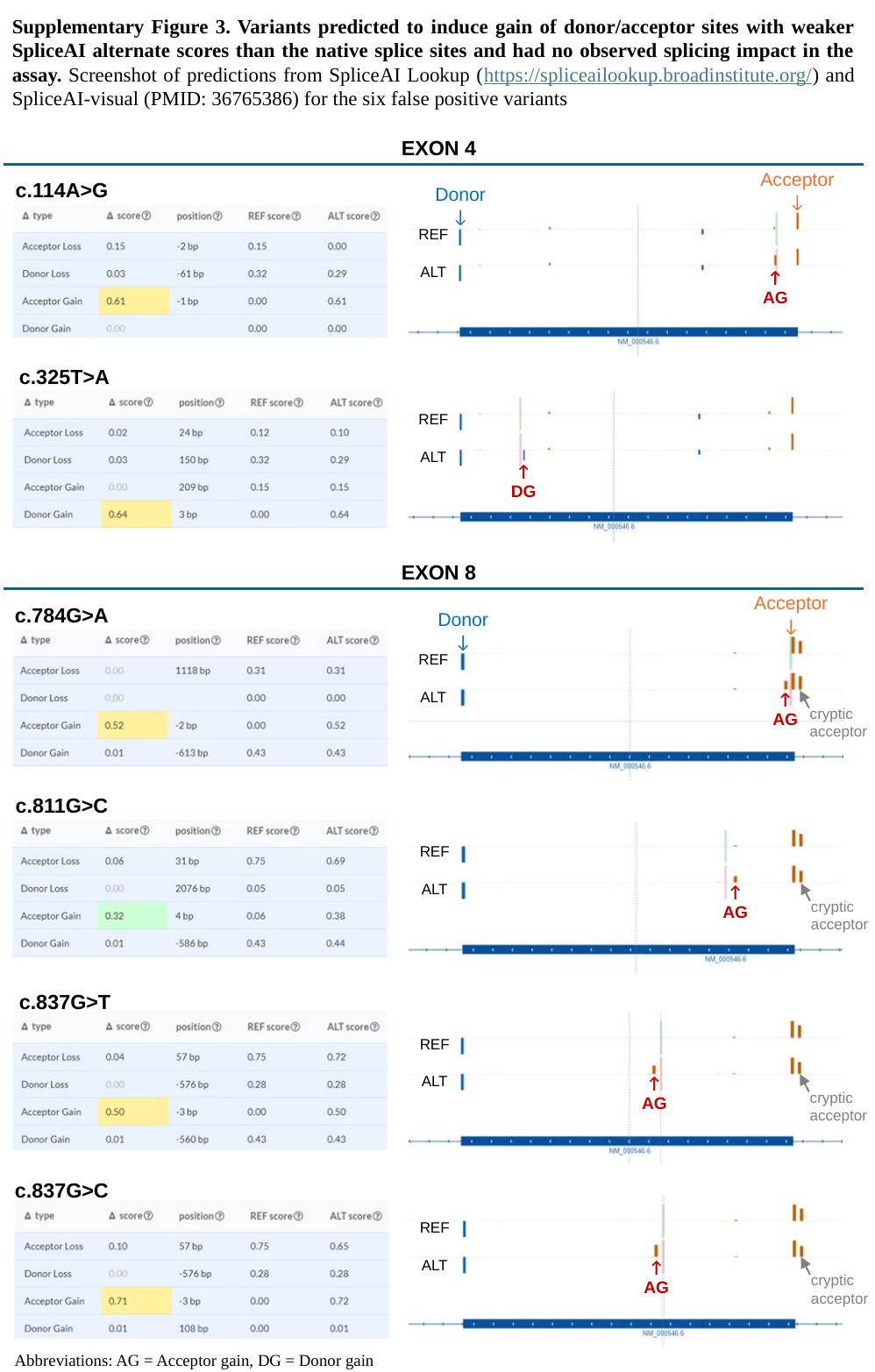

Supplementary Figure 3. Variants predicted to induce gain of donor/acceptor sites with weaker SpliceAI alternate scores than the native splice sites and had no observed splicing impact in the assay. Screenshot of predictions from SpliceAI Lookup (https://spliceailookup.broadinstitute.org/) and SpliceAI-visual (PMID: 36765386) for the six false positive variants
EXON 4
Acceptor
↓
c.114A>G
REF
ALT
Donor
↓
↑
AG
c.325T>A
REF
ALT
↑
DG
EXON 8
Acceptor
↓
c.784G>A
REF
ALT
↑
AG
cryptic acceptor
Donor
↓
c.811G>C
REF
ALT
↑
AG
cryptic acceptor
c.837G>T
REF
ALT
↑
AG
cryptic acceptor
c.837G>C
REF
ALT
↑
AG
cryptic acceptor
Abbreviations: AG = Acceptor gain, DG = Donor gain
